# The mRNA Cap 2′-*O*-Methyltransferase CMTR1 Regulates the Expression of Certain Interferon-Stimulated Genes

**DOI:** 10.1128/mSphere.00202-20

**Published:** 2020-05-13

**Authors:** Graham D. Williams, Nandan S. Gokhale, Daltry L. Snider, Stacy M. Horner

**Affiliations:** a Department of Molecular Genetics and Microbiology, Duke University Medical Center, Durham, North Carolina, USA; b Department of Medicine, Duke University Medical Center, Durham, North Carolina, USA; University of Texas Southwestern Medical Center

**Keywords:** ISGs, RNA modification, antiviral, interferon

## Abstract

Type I interferons (IFN) initiate an antiviral state through a signal transduction cascade that leads to the induction of hundreds of IFN-stimulated genes (ISGs) to restrict viral infection. Recently, RNA modifications on both host and viral RNAs have been described as regulators of infection. However, the impact of host mRNA cap modifications on the IFN response and how this regulates viral infection are unknown. Here, we reveal that CMTR1, an ISG that catalyzes 2′-*O*-methylation of the first transcribed nucleotide in cellular mRNA (Cap 1), promotes the protein expression of specific ISGs that contribute to the antiviral response. Depletion of CMTR1 reduces the IFN-induced protein levels of ISG15, MX1, and IFITM1, without affecting their transcript abundance. However, CMTR1 depletion does not significantly affect the IFN-induced protein or transcript abundance of IFIT1 and IFIT3. Importantly, knockdown of IFIT1, which acts with IFIT3 to inhibit the translation of RNAs lacking Cap 1 2′-*O*-methylation, restores protein expression of ISG15, MX1, and IFITM1 in cells depleted of CMTR1. Finally, we found that CMTR1 plays a role in restricting RNA virus replication, likely by ensuring the expression of specific antiviral ISGs. Taken together, these data reveal that CMTR1 is required to establish an antiviral state by ensuring the protein expression of a subset of ISGs during the type I IFN response.

**IMPORTANCE** Induction of an efficient type I IFN response is important to control viral infection. We show that the host 2′-*O*-methyltransferase CMTR1 facilitates the protein expression of ISGs in human cells by preventing IFIT1 from inhibiting the translation of those mRNAs lacking cap 2′-*O*-methylation. Thus, CMTR1 promotes the IFN-mediated antiviral response.

## Abstract (precis)

Induction of an efficient type I IFN response is important to control viral infection. We show that the host 2′-*O*-methyltransferase CMTR1 facilitates the protein expression of ISGs in human cells by preventing IFIT1 from inhibiting the translation of those mRNAs lacking cap 2′-*O*-methylation. Thus, CMTR1 promotes the IFN-mediated antiviral response.

## INTRODUCTION

Interferons (IFN) are cytokines that establish an antiviral cellular state through transcriptional induction of interferon-stimulated genes (ISGs) (1). Following engagement of IFN (type I and type III) with its cognate cell surface receptors, a JAK-STAT signaling cascade is activated that results in the formation of the ISGF3 transcription factor complex that binds to IFN response elements in the promoters of ISGs. Following their transcription, ISG mRNAs are translated, generating the proteins that restrict viral infection and viral spread (2, 3). Full induction of this antiviral state requires hundreds of ISGs to be upregulated efficiently; therefore, cells must encode mechanisms to ensure their regulated expression. Such regulation can occur at both the transcriptional and posttranscriptional levels (4–6). While several posttranscriptional controls of antiviral cytokines and signaling molecules have been defined, similar mechanisms that regulate ISG expression have largely been left unexplored (5, 7).

**FIG 1:**
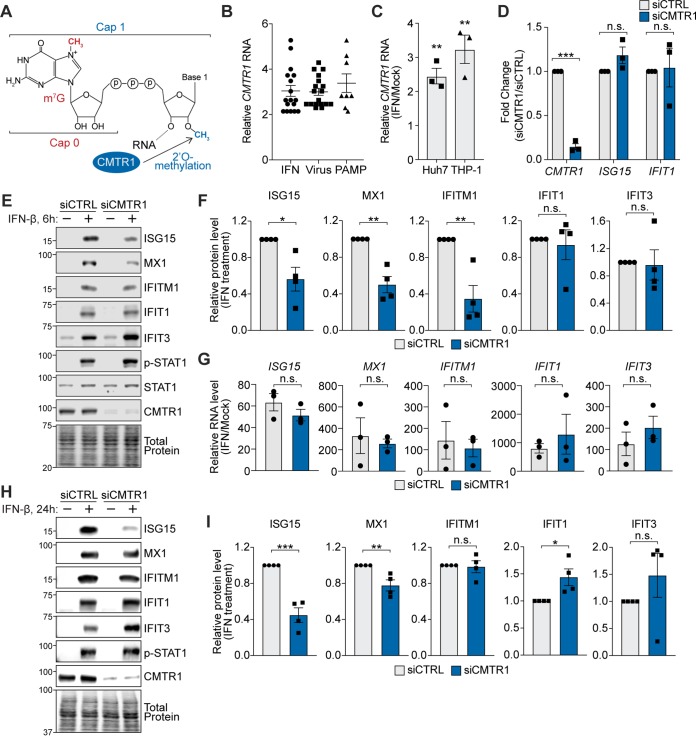
CMTR1 promotes the protein expression of specific ISGs. (A) Schematic of 5′ cap structure, showing m^7^G (Cap 0) and 2′-*O*-methylation of the first transcribed nucleotide catalyzed by CMTR1 (Cap 1). (B) Fold induction of *CMTR1* treated with the indicated stimuli, including IFN (type I, type II, or type III), viruses (human rhinovirus 16, Newcastle disease virus, human cytomegalovirus strain TB40E, influenza A virus, and yellow fever virus 17D), and pathogen-associated molecular patterns [PAMP; lipopolysaccharide or poly (I·C)], relative to untreated samples. Data were obtained from the EMBL-EBI Expression Atlas RNA-seq database (34). (C) RT-qPCR analysis of *CMTR1* relative to *GAPDH* in either Huh7 or THP-1 cells following IFN-β treatment, compared to the levels in untreated cells. (D) RT-qPCR analysis of *CMTR1*, *ISG15*, and *IFIT1* relative to *GAPDH* in mock-treated Huh7 cells transfected with the indicated siRNAs. (E) Representative immunoblot of extracts from mock-treated or IFN-β-treated (6 h) Huh7 cells transfected with the indicated siRNAs. (F) Quantification of results from the immunoblots shown in panel E, normalized to total protein and graphed relative to the siRNA control (siCTRL). (G) RT-qPCR analysis of *ISG* genes relative to *GAPDH* in IFN-β-treated Huh7 cells transfected with the indicated siRNAs. Data are graphed as fold changes relative to mock-treated cells. (H) Representative immunoblot of extracts from mock- or IFN-β-treated (24 h) Huh7 cells transfected with the indicated siRNAs. (I) Quantification of the immunoblots from panel H normalized to total protein and graphed relative to siCTRL. All IFN-β treatments were performed at 50 U/ml for the indicated times. Values represent means ± standard errors of the means (SEM) of results from 3 (C, D, and G) or 4 (F and I) biological replicates. *, *P* < 0.05; **, *P* < 0.01; ***, *P* < 0.001 (unpaired Student's *t* test). n.s., not significant.

Chemical modifications at the 5′ end of mRNAs are required for optimal gene expression (8). One such modification is N^7^-methylguanosine (m^7^G), which is added cotranscriptionally to the first transcribed nucleotide at the 5′ end of mRNAs (referred to as Cap 0; Fig. 1A). In higher eukaryotes, the ribose 2′-*O*-hydroxyl group of the first transcribed nucleotide is methylated, resulting in the Cap 1 structure (Fig. 1A) (8). Approximately 50% of cellular mRNAs are also methylated at the ribose 2′-*O*-hydroxyl group of the second transcribed nucleotide (9). These mRNA cap modifications have been shown to have a wide variety of functions. For example, the m^7^G modification in the mRNA cap recruits the translation initiation machinery for cap-dependent translation, and Cap 1 2′-*O*-methylation can further enhance this translation (8, 10, 11). mRNA cap 2′-*O*-methylation has also been shown to prevent degradation of modified transcripts by exoribonucleases or to serve as a motif that marks mRNA as “self” to prevent recognition by the IFN-induced RNA binding proteins RIG-I and IFIT1 (12–15). RIG-I binding to Cap 0 mRNA can activate a signaling cascade that induces an IFN response (12, 16), whereas IFIT1 binding to Cap 0 mRNA inhibits its translation (13, 17–19). This translational inhibition by IFIT1 is in part mediated by its interaction with the IFN-induced protein IFIT3, which increases the specificity of IFIT1 for Cap 0 mRNAs (20–22). As such, many viruses have evolved mechanisms to evade RIG-I and IFIT1 sensing of their 5′ RNA ends. Some examples of these viral strategies to evade RIG-I and IFIT1 sensing include encoding proteins that bind to and shield Cap 0 in viral mRNA; acquiring Cap 1 from cellular mRNAs; altering their RNA structures at the 5′ end; and encoding their own viral Cap 1 2′-*O*-methyltransferases (8, 23–30), highlighting the overall importance of this RNA modification in “self versus nonself” recognition.

Cap 1 2′-*O*-methylation of host transcripts is catalyzed by the cellular enzyme CMTR1 (31, 32). Interestingly, CTMR1, also called ISG95, is transcriptionally induced following induction of IFN (32, 33). Therefore, we hypothesized that CMTR1 may be required to promote the IFN-mediated induction of ISGs and the antiviral response. Here, we demonstrate that CMTR1 is required for full expression of specific ISGs in response to type I IFN. We found that depletion of CMTR1 results in decreased protein production of certain ISGs without affecting the abundance or stability of their associated transcripts. Importantly, this reduced ISG expression in the absence of CMTR1 leads to increased virus replication. In addition, we found that the inhibition of protein production of the CMTR1-regulated ISGs is mediated by the 2′-*O*-methylation sensor IFIT1, as loss of IFIT1 rescued their protein expression following CMTR1 depletion. Together, these results reveal that CMTR1 is required for efficient expression of ISGs and the resulting antiviral state.

## RESULTS

### CMTR1 promotes the protein expression of specific ISGs.

Previous studies have shown that CMTR1 is an ISG that is upregulated in response to multiple stimuli, including type I and type II IFN, as well as viral infection and multiple pathogen-associated molecular patterns (PAMPs) which induce IFN (Fig. 1B) (32–34). We confirmed that the *CMTR1* transcript is induced in response to beta IFN (IFN-β) in human Huh7 liver hepatoma cells and human monocyte THP-1 cells, as measured by reverse transcription-quantitative PCR (RT-qPCR) (Fig. 1C). Because of the previously established role of CMTR1 in regulating gene expression, we hypothesized that it may regulate the expression of other genes induced by IFN. To test this, we depleted CMTR1 using small interfering RNAs (siRNAs) and then measured the transcript levels and protein expression of a set of ISGs in the presence or absence of IFN-β. While others have shown that CMTR1 depletion induced *IFIT1* in the absence of exogenous IFN-β in primary human fibroblasts, we found that in unstimulated Huh7 cells, CMTR1 depletion did not alter ISG transcripts, indicating the CMTR1 does not affect basal IFN-β and ISG expression (Fig. 1D) (16). However, we did find that following IFN-β treatment for 6 h, CMTR1 depletion resulted in decreased protein expression of the ISGs ISG15, MX1, and IFITM1, while the levels of expression of the ISGs IFIT1 and IFIT3 remained largely unaffected (Fig. 1E and F). We also found that the relative RNA levels of these ISGs in response to IFN-β in Huh7 cells were not altered by CMTR1 depletion, as measured by RT-qPCR (Fig. 1G). Interestingly, following 24 h of IFN-β stimulation, CMTR1 depletion resulted in decreased protein expression of ISG15 and MX1, while the expression of IFITM1 was no longer decreased (Fig. 1H and I). These data reveal that CMTR1 promotes the rapid protein expression of certain ISGs in response to IFN-β, without affecting expression of their mRNAs.

### CMTR1 is required for the antiviral response.

**FIG 2:**
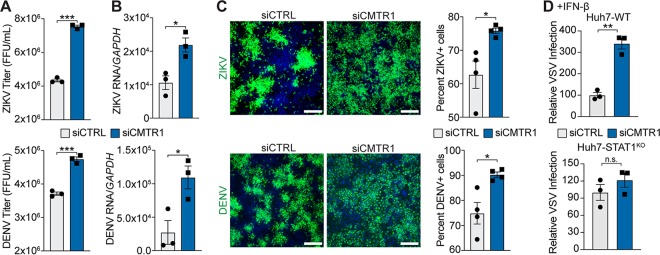
CMTR1 is required for the antiviral response. (A) Focus-forming assay (FFU) of supernatants. (B) RT-qPCR analysis of viral RNA, relative to *GAPDH*, harvested from ZIKV- or DENV-infected Huh7 cells (48 h postinfection [hpi]; multiplicity of infection [MOI] of 0.01) treated with the indicated siRNAs. (C) (Left) Representative fields of ZIKV- and DENV-infected Huh7 cells (48 hpi; MOI 0.01) treated with the indicated siRNAs and immunostained with anti-flaviviral E protein (green). Nuclei were stained with DAPI (blue). Scale bar: 100 μm. (Right) Quantification of the percentages of ZIKV- and DENV-infected Huh7 cells. (D) Quantification of the relative numbers of VSV-GFP-positive Huh7 wild-type (WT) and STAT1^KO^ cells after siRNA treatment and IFN-β pretreatment (25 U/ml; 16 h) determined at 8 hpi and set to a value of 100. For panels C and D, ≥5,000 cells were counted in each experiment per condition. Values represent means ± SEM of results from 3 biological replicates. *, *P* < 0.05; **, *P* < 0.01; ***, *P* < 0.001 (unpaired Student's *t* test). n.s., not significant.

Infection of human cells by RNA viruses is often restricted by the antiviral functions of the proteins encoded by ISGs (2, 3). Because CMTR1 is required for the expression of specific ISGs, we hypothesized that it would also regulate the antiviral response to IFN-sensitive viruses, such as the positive-sense RNA viruses Zika virus (ZIKV) and dengue virus (DENV) (35). Importantly, since both of these viruses encode their own Cap 1 2′-*O*-methyltransferase activity, they are not reliant on CMTR1 for 2′-*O*-methylation of their mRNA (36). At 48 h postinfection with either ZIKV or DENV, we measured virus replication by using three complementary assays: the production of infectious virus in the supernatant by focus-forming assay, viral RNA replication by RT-qPCR, and the percentage of infected cells by immunofluorescence microscopy. We found for both ZIKV and DENV that depletion of CMTR1 resulted in higher levels of infectious virus, higher levels of viral RNA, and higher percentages of infected cells than were seen with the control cells (Fig. 2A to C). Further, depletion of CMTR1 resulted in increased viral spread compared to control cells, in which viral replication was limited to discrete foci (Fig. 2C). To determine directly whether CMTR1 contributed to viral restriction by type I IFN, we measured the ability of type I IFN to restrict infection by another IFN-sensitive virus, vesicular stomatitis virus (VSV), a negative-sense RNA virus which also encodes its own Cap 1 2′-*O*-methyltransferase (37–39), following CMTR1 depletion. CMTR1 depletion resulted in an increase in the percentage of VSV-infected cells in IFN-β-pretreated Huh7 cells compared to control cells. Importantly, this increase was not seen in the absence of STAT1, which is required for IFN-mediated induction of ISGs (Fig. 2D). Together, these results suggest that CMTR1 is required to facilitate IFN-mediated induction of the cellular antiviral state.

### CMTR1 does not regulate nuclear export, RNA stability, or polysome association of specific ISGs.

**FIG 3:**
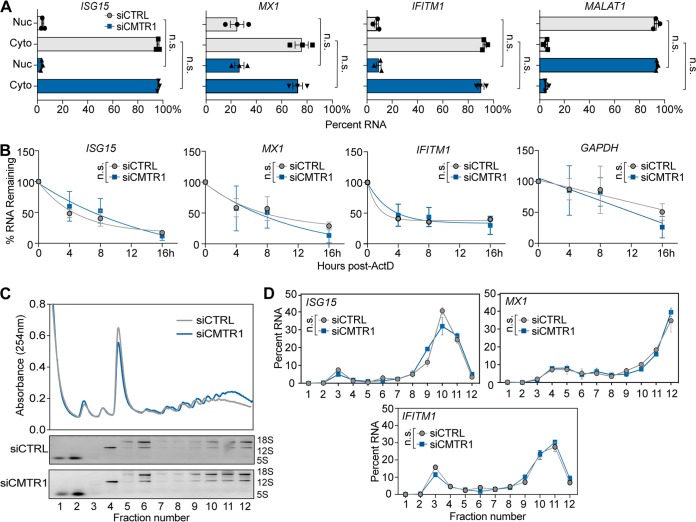
CMTR1 does not regulate nuclear export, RNA stability, or polysome association of specific ISGs. (A) Relative percentages of *ISG* genes or *MALAT1* (nuclear fractionation control) in nuclear (Nuc) and cytoplasmic (Cyto) fractions of siRNA-treated and IFN-β-treated Huh7 cells, as analyzed by RT-qPCR. (B) RNA stability, as measured by the percentages of transcript remaining in siRNA-treated and IFN-β-treated Huh7 cells at the indicated times following actinomycin D (10 μg/ml) treatment. (C) (Top) Representative plot of the relative absorbance values of fractions isolated from extracts of siRNA-treated and IFN-β-treated Huh7 cells following centrifugation over 15 to 50% sucrose gradients. (Bottom) RNA from each fraction was separated on an agarose gel and visualized with ethidium bromide. rRNA bands (18S, 12S, and 5S) are indicated. (D) RT-qPCR analysis of ISGs from polysome profiling of the extracts described in the panel C legend. Data are presented as the percentage of total mRNA in each fraction. All IFN-β treatments were performed at 50 U/ml for 6 h. Values represent means ± SEM of results from 3 biological replicates. Data were analyzed by (A) ordinary one-way analysis of variance (ANOVA) with Sidak′s multiple-comparison test or by (B and D) the Holm-Sidak method, with an alpha value of 0.05 and each time point or fraction analyzed individually without assuming a consistent standard deviation. n.s., not significant.

As we found that CMTR1 depletion resulted in lowered protein levels but not in lowered mRNA expression of specific ISGs following IFN-β stimulation (6 h; ISG15, MX1, and IFITM1), we sought to determine the molecular mechanism underlying this change in protein expression. Cap 1 has been shown to shield mRNAs from degradation, regulate mRNA nuclear export, and promote mRNA translation (8, 9, 14, 15, 40). Therefore, we tested how loss of CMTR1 regulated these processes for ISG15, MX1, and IFITM1. Following IFN-β treatment, neither the nuclear export of these ISGs nor their stability was altered by CMTR1 depletion (Fig. 3A and B). To determine whether CMTR1 depletion affected mRNA translation, we performed polysome analysis of IFN-β-treated Huh7 cells. We found no differences in the overall polysome profiles of these cells following CMTR1 depletion compared to control cells (Fig. 3C). Surprisingly, we also found no difference in the polysome association of the CMTR1-regulated ISG transcripts (*ISG15*, *MX1*, and *IFITM1*) following CMTR1 depletion (Fig. 3D). Collectively, these results suggest that loss of CMTR1 does not globally alter cellular translation in response to IFN-β, which is similar to results described previously by others (41), and does not alter nuclear export, transcript stability, or polysome association of the CMTR1-regulated ISGs.

### CMTR1 inhibits IFIT1-mediated translational regulation of specific ISGs via their 5′ untranslated regions (5′ UTRs).

**FIG 4:**
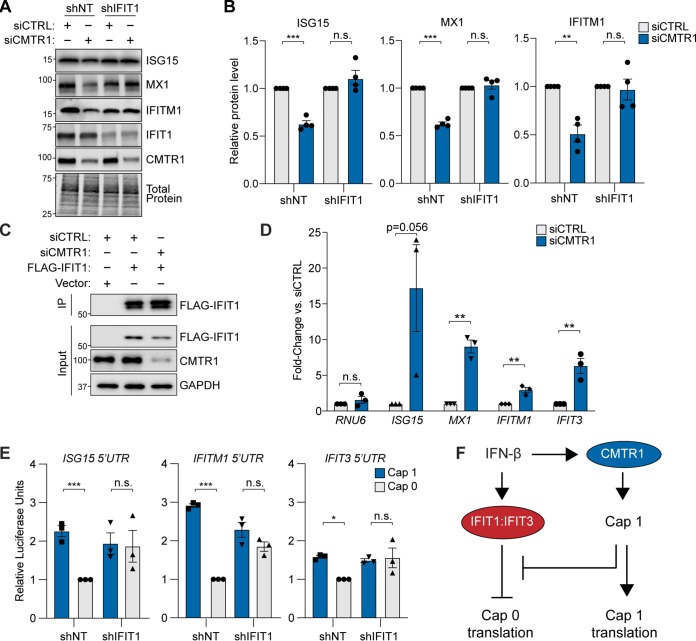
CMTR1 inhibits IFIT1-mediated translational regulation of specific ISGs via their 5′ UTRs. (A) Representative immunoblot of extracts of IFN-β-treated Huh7 cells transduced with the indicated shRNA (nontargeting control [NT] and IFIT1) and then transfected with siCTRL or siCMTR1. (B) Quantification of immunoblots from the experiment described in the panel A legend. The values represent the relative protein levels of the indicated ISGs normalized to the total protein levels, which were then set to a value of 1 for each set of siCTRL samples. The data represent means ± SEM of results from 4 biological replicates. (C) Representative immunoblot of FLAG-immunoprecipitated (IP) or input fractions of IFN-β-treated Huh7 cells transfected with vector or FLAG-IFIT1, as well as siCTRL or siCMTR1. (D) RT-qPCR analysis of enriched transcripts relative to input by anti-FLAG IP in siRNA-treated and IFN-β-treated Huh7 cells. Fold enrichment values for each transcript are graphed relative to siCTRL and represent means ± SEM of results from 3 biological replicates. (E) Secreted *Gaussia* luciferase units corresponding to a reporter whose expression was driven by the 5′ UTR of the indicated ISGs and contained either Cap 0 or Cap 1 in IFN-β-treated Huh7 cells transduced with shNT or shIFIT1. Relative fold change values for the indicated reporters are shown, with the shNT cells transfected with the indicated Cap 0 construct set to a value of 1 for each experiment; samples representing that RNA were normalized to this value. These values were then graphed as means ± SEM of results from 3 biological replicates. (F) Following IFN-β-signaling, IFIT1 and IFIT3 were induced, and they were found to be able to inhibit the translation of Cap 0 mRNAs. However, CMTR1 induction by IFN-β can ensure that the mRNA of ISGs is modified by Cap 1, ensuring ISG protein expression and the antiviral response. **, *P* < 0.01; ***, *P* < 0.005 (unpaired Student's *t* test) (B and D); *, *P* < 0.05; ***, *P* < 0.005 (ordinary one-way ANOVA with Sidak′s multiple-comparison test) (E). n.s., not significant. All IFN-β treatments were performed at 50 U/ml for 6 h.

mRNAs that lack Cap 1 2′-*O*-methylation are known to be preferentially bound by IFIT1 (13, 19–22, 25, 27, 30, 42), and translation of viral RNAs lacking Cap 1 2′-*O*-methylation is known to be inhibited by IFIT1 (20, 21, 42). Therefore, we hypothesized that the protein expression of host transcripts lacking Cap 1 2′-*O*-methylation may be inhibited by IFIT1. Indeed, we found that depletion of IFIT1 rescued the protein expression of ISG15, MX1, and IFITM1, resulting in equivalent levels of expression of ISGs following CMTR1 depletion compared to control results in IFN-β-treated Huh7 cells (Fig. 4B). We did not examine IFIT3 in this assay, as it is in a complex with IFIT1; alterations in IFIT1 levels likely also alter IFIT3 expression (20, 21, 43). Therefore, the translational repression of the CMTR1-dependent ISGs is mediated through the actions of IFIT1.

To determine how IFIT1 mediates this repression of protein expression following CMTR1 depletion, we first tested if IFIT1 bound selectively to the transcripts of the CMTR1-regulated ISGs. However, we found that IFIT1 bound to both the CMTR1-regulated and the CMTR1-nonregulated transcripts following CMTR1 depletion (Fig. 4C and D). As a control, we tested IFIT1 binding to the U6 snRNA (*RNU6*), which has a gamma-monomethyl 5′ cap, and we found, similarly to results reported previously by others (44), that IFIT1 did not bind this RNA. We next tested if CMTR1-regulated ISGs contained RNA elements that could dictate their sensitivity to translational repression by IFIT1. We generated reporters in which the expression of *Gaussia* luciferase was driven by the 5′ UTRs of either CMTR1-regulated *ISG15* and *IFITM1* or non-CMTR1-regulated *IFIT3.* We used *in vitro* transcription to generate reporter transcripts and then added either the m^7^G modification alone (Cap 0) or both m^7^G and 2′-*O*-methylation of the ribose (Cap 1) to the first transcribed nucleotide. These transcripts containing different 5′ UTRs and cap structures were then transfected into IFN-β-primed Huh7 cells. At 6 h posttransfection, we measured the luciferase activity of these reporters and found that the reporters containing the 5′ UTRs of the CMTR1-regulated ISGs *ISG15* and *IFITM1* showed luciferase activity that was decreased (about 2-fold to 3-fold) in the absence of 2′-*O*-methylation in the cap (Cap 0), while the luciferase activity of the reporter containing the 5′ UTR of the CMTR1-nonregulated ISG *IFIT3* was only modestly altered (decreased 1.5-fold) by lack of Cap 1 2′-*O*-methylation (Fig. 4E). Additionally, we found that when IFIT1 was depleted from these cells, the luciferase activity of these reporters was no longer altered by lack of Cap 1 2′-*O*-methylation (Fig. 4E). Thus, these results reveal that the presence of Cap 1 within the 5′ UTRs of CMTR1-regulated ISGs alleviates the translational inhibition mediated by IFIT1 during the IFN response (Fig. 4F). Further, these results reveal that the 5′ UTR of the non-CMTR1-regulated ISG IFIT3 contains an RNA element(s) that allows it to partially overcome IFIT1-mediated translational inhibition.

## DISCUSSION

We hypothesized that ISGs induced during the IFN response would be reliant on Cap 1 2′-*O*-methylation by CMTR1 for evasion of IFIT1 translational inhibition. Here, we show that the protein expression of specific ISGs (ISG15, IFITM1, and MX1) is enhanced by CMTR1, while the expression of the ISGs IFIT1 and IFIT3, which suppress translation of both viral and cellular mRNAs lacking Cap 1 2′-*O*-methylation (13, 18–22, 25, 27, 30, 45–47), is not as sensitive to CMTR1 levels. Surprisingly, while IFIT1 bound to all tested ISGs when CMTR1 was depleted, only the protein expression of the CMTR1-regulated ISGs was inhibited by IFIT1 under these conditions. Importantly, we found that the 5′ UTR of *IFIT3* was not as reliant as the 5′ UTRs of *ISG15* and *IFITM1* on Cap 1 2′-*O*-methylation to prevent IFIT1-mediated translational regulation. Therefore, these data reveal that during the type I IFN response, the mRNAs of some ISGs require CMTR1 to evade sensing and translational inhibition by IFIT1 and that other ISGs encode additional mechanisms to evade this sensing. Ultimately, this suggests that during the IFN response, Cap 1 2′-*O*-methylation of transcripts by CMTR1 ensures their protein expression to program a functional IFN response.

We found that CMTR1, by preventing IFIT1-mediated repression, is required for the protein expression of ISGs in response to IFN. Our data supports the conclusion that CMTR1 depletion does not affect IFN expression but that it instead specifically regulates the protein expression of certain ISGs. If the production of IFN were decreased by CMTR1 depletion, then we would expect that the IFN-mediated induction of all ISGs would be reduced at the mRNA level following CMTR1 depletion. However, the relative levels of IFN-mediated mRNA induction of 5 different ISGs were similar in the presence and absence of CMTR1 (Fig. 1G). Further, while others have reported that loss of CMTR1 and/or Cap 1 2′-*O*-methylation actually triggers RIG-I sensing of host mRNAs to induce expression of IFN and ISGs under certain conditions (12, 16), we did not observe this upon CMTR1 depletion in Huh7 cells (Fig. 1D). This allowed us to focus on how changes in CMTR1 levels influence ISG protein expression through IFIT1 restriction of RNAs lacking Cap 1 2′-*O*-methylation. While the molecular mechanisms by which IFIT1 inhibits or, more likely, delays the translation of RNAs lacking Cap 1 2′-*O*-methylation are not fully clear (13, 18–22, 29, 45–47), it has been shown that IFIT1 can compete with the cap binding protein eukaryotic initiation factor 4 subunit E (eIF4E) and also with eIF4F for binding to Cap 0 RNAs and prevent formation of the preinitiation complex (19, 22, 46, 47). Further, it has been shown that murine IFIT1 decreases the translation rate of viral RNAs lacking Cap 1 (19). To date, no study has shown the effects of IFIT1 on the polysome occupancy of its regulated transcripts. Indeed, we did not observe a change in the polysome occupancy of the IFIT1-regulated ISGs upon CMTR1 depletion, even though the reduction in the corresponding levels of protein expression seen upon CMTR1 depletion or as a consequence of loss of Cap 1 2′-*O*-methylation was mediated by IFIT1 (Fig. 4). While we do not yet know the mechanism of IFIT1-mediated translational repression, our data support the conclusion that IFIT1 inhibits the translation of CMTR1-regulated ISGs.

Interestingly, the levels of protein expression of IFIT1 and IFIT3 were not significantly affected by CMTR1 depletion, suggesting that these mRNAs encode a mechanism to overcome the restriction that resulted in reduced protein expression of ISG15, MX1, and IFITM1 following CMTR1 depletion. Originally, we hypothesized that the reason that the levels of IFIT1 and IFIT3 protein expression were not affected by CMTR1 depletion was that these mRNAs were not bound by IFIT1 in the absence of CMTR1. However, we found that *IFIT3* mRNA was still bound by IFIT1 in the absence of CMTR1 (Fig. 4). Therefore, we next tested if the 5′ UTR encoded elements of ISGs affected IFIT1-mediated translational inhibition and CMTR1 dependence. Indeed, we found that when IFIT1 was upregulated by IFN treatment, the translation of a luciferase reporter driven by the 5′ UTR of *IFIT3* (not regulated by CMTR1) was only partially inhibited by the absence of Cap 1 2′-*O*-methylation, while the translation of reporters with the 5′ UTR of either *ISG15* or *IFITM1* (regulated by CMTR1) was more strongly inhibited by the absence of Cap 1 2′-*O*-methylation (Fig. 4). This suggests three possible mechanisms underlying how the CMTR1 sensitivity of these ISGs is regulated. First, it is possible that features in the 5′ UTR of the non-CMTR1-regulated ISG IFIT3 prevent IFIT1 translational inhibition. In support of this idea, the 5′ UTR of some viral RNAs can encode secondary structures that prevent IFIT1 sensing and translational inhibition (18, 30, 42). Alternatively, the non-CMTR1-regulated ISG IFIT3 may have only low levels of Cap 1 and, if so, may not be as sensitive to the changes in CMTR1 and the resulting decrease in Cap 1 levels that would enable IFIT1 sensing and translational inhibition. Indeed, CMTR1 has reduced activity on mRNAs with highly structured 5′ UTRs and, as such, these structured 5′ UTRs may require unwinding by the helicase DHX15 for methylation by CMTR1. Interestingly, DHX15 activity is increased by CMTR1 binding (41, 48). Therefore, CMTR1 likely acts in concert with DHX15 to regulate the expression of certain ISGs. Finally, it is possible that CMTR1 depletion preferentially affects the levels of Cap 1 in ISGs with highly structured 5′ UTRs which are not efficiently 2′-*O*-methylated, thereby specifically resulting in reduced translation of these transcripts following CMTR1 depletion. In any case, our results suggest that RNA features in ISG transcripts can influence IFIT1 translational regulation. Future work should determine the complete repertoires of CMTR1-dependent and -independent cellular RNAs during the IFN response to define novel elements that mediate the function of IFIT1. Overall, this work reveals that CMTR1 promotes the protein expression of specific ISGs to efficiently establish an IFN-dependent antiviral state.

## MATERIALS AND METHODS

### Cell culture, viral stocks, and viral infection.

Huh7, 293T, and Vero cells were grown in Dulbecco′s modification of Eagle′s medium (DMEM; Mediatech) supplemented with 10% fetal bovine serum (FBS; HyClone), 25 mM HEPES (Thermo Fisher), and 1× nonessential amino acids (Gibco) (referred to here as cDMEM). THP1 cells (a gift from Dennis Ko, Duke University, who obtained them from the American Type Culture Collection [ATCC]) were grown in RPMI 1640 medium (RPMI 1640; Thermo Fisher) supplemented with 10% FBS and 25 mM HEPES. All cell lines were grown at 37°C with supplementation with 5% CO_2_. 293T and Vero (CCL-3216 and CCL-81) cells were obtained from ATCC; Huh7 cells were a gift of Michael Gale, Jr. (University of Washington); Huh7-STAT1^KO^ (Huh7-STAT1 knockout) cells have been described previously (49). To generate short hairpin RNA (shRNA) IFIT1 cells, 293T cells were cotransfected with psPax2 and pMD2.G (Addgene plasmids; catalog no. 12260 and 12259), as well as pLKO.1 (50) containing either nontargeting (NT) or IFIT1 shRNA (Sigma; TRC1, clone TRCN0000158439). Huh7 cells were transduced with the indicated shRNA expressing lentivirus and then selected with 2 μg/ml puromycin (Sigma). After selection, cells were maintained in 1 μg/ml puromycin until use. The identity of cell lines was verified using a Promega GenePrint STR kit (DNA Analysis Facility, Duke University), and cells were verified as mycoplasma free by the use of a LookOut Mycoplasma PCR detection kit (Sigma).

Infectious stocks of a cell culture-adapted strain of DENV2-NGC and ZIKV-DAKAR were generated and titers were determined as described previously (51). ZIKV-DAKAR (Zika virus/A,africanus-tc/SEN/1984/41525-DAK) was provided by Scott Weaver (University of Texas Medical Branch). DENV2-NGC and ZIKV-DAKAR infections (multiplicity of infection [MOI] 0.01) were performed in Huh7 cells in serum-free media for 4 h, after which cDMEM was replenished for a total infection time of 48 h. VSV-GFP (VSV-green fluorescent protein), provided by Sean Whelan (Harvard University), was propagated in Vero cells, as described previously (39). Infections (MOI 0.2) were performed in Huh7 cells in serum-free media for 4 h, after which cDMEM was replenished for a total infection time of 8 h. Sendai virus (SenV) strain Cantell was obtained from Charles River Laboratories and used at 200 hemagglutination units/ml. SenV infections were performed in serum-free media for 1 h, after which cDMEM was replenished.

### Plasmids.

The following plasmids used in this study have been previously described: pEF-Tak-Flag (52) and pCMV-Gluc2 (NEB). The following plasmids were generated in this study: pEF-Tak-Flag-IFIT1, pCMV-5′UTR ISG15 TATT, pCMV-5′UTR IFITM1 TATT, and pCMV-5′UTR IFIT3. To generate pEF-Tak-Flag-IFIT1, HEK293T cells were stimulated with SenV for 24 h, cDNA was created using SuperScript III (Thermo Fisher), and then IFIT1 was amplified by PCR. The IFIT1 amplicon was inserted into NotI-PmeI-digested pEF-Tak-Flag vector by InFusion cloning (Clontech). The following 5′ UTR constructs were purchased from IDT as gBlocks: ISG15 (RefSeq accession no. NM_005101.4; UTRDB accession no. 5HSAR051845), IFITM1 (RefSeq accession no. NM_003641.4; UTRDB accession no. 5HSAR055561), and IFIT3 (RefSeq accession no. NM_001549.6; UTRDB accession no. 5HSAR021528) (53) (http://utrdb.ba.itb.cnr.it). The constructs were inserted into HindIII-BamHI digested pCMV-Gluc2 by InFusion cloning. This resulted in pCMV-5′ UTR IFIT3. For ISG15 and IFITM1, the final nucleotide of the TATA box of pCMV-Gluc2 was changed to a T by using site-directed mutagenesis (QuikChange Lightning; Agilent) to facilitate *in vitro* transcription (54), resulting in pCMV-5′UTR ISG15 TATT and pCMV-5′UTR IFITM1 TATT. All DNA sequences were verified by sequencing. Primer sequences are provided in Table 1.

**TABLE 1:** Primers used for cloning and RT-qPCR

Primer name	Primer sequence
*GAPDH* F	5′-AAGGTGAAGGTCGGAGTCAAC
*GAPDH* R	5′-GGGGTCATTGATGGCAACAATA
*IFIT1* F	5′-TCCTTGGGTTCGTCTACAAAT
*IFIT1* R	5′-TTCTCAAAGTCAGCAGCCAGT
*ISG60* F	5′-AGTCTAGTCACTTGGGGAAAC
*ISG60* R	5′-ATAAATCTGAGCATCTGAGAGTC
*ISG15* F	5′-GCGAACTCATCTTTGCCAGTA
*ISG15* R	5′-CCAGCATCTTCACCGTCAG
*IFITM1* F	5′-ACTAGTAGCCGCCCATAGCC
*IFITM1* R	5′-GCACGTGCACTTTATTGAATG
*MXA* F	5′-TTCAGCACCTGATGGCCTATC
*MXA* R	5′-TGGATGATCAAAGGGATGTGG
*CMTR1* F	5′-CGAACTTCTTTGAGCTAATCCAG
*CMTR1* R	5′-CAGCGGTAGTCAAACACAGG
*MALAT1* F	5′-GACGGAGGTTGAGATGAAGC
*MALAT1* R	5′-ATTCGGGGCTCTGTAGTCCT
*RNU6* F	5′-GCTTCGGCAGCACATATACTAAAATTGGA
*RNU6* R	5′-ATAGGAACGCTTCACGAATTTGCG
*DENV* F	5′-GAAACGCGAGAGAAACCGCG
*DENV* R	5′-CGCCACCAGGGCCATGAACAG
*ZIKV* F	5′-TAGAGGAATGGTGCTGTAGGGA
*ZIKV* R	5′-CTGGTTCCTTTCTGGGCCTTAT
*Gluc* F	5′-CGACATTCCTGAGATTCCTGG
*Gluc* R	5′-TTGAGCAGGTCAGAACACTG
ISG15_TATT_F	5′-GCAGCACCGGCCCTATTATAATAGTGAGTCGTATTAATT
ISG15_TATT_R	5′-AATTAATACGACTCACTATTATAATAGGGCCGGTGCTGC
IFITM1_TATT_F	5′-CTATTTCCTGCTGTTTAATAGTGAGTCGTATTAATTTCGATAAGCC
IFITM1_TATT_R	5′-GGCTTATCGAAATTAATACGACTCACTATTAAACAGCAGGAAATAG
5′UTR F	5′-CGAAATTAATACGACTCAC
5′UTR R	5′-GGGCAAACAGAACTTTGAC

### *In vitro* transcription and mRNA capping.

Purified NotI-linearized pCMV-Gluc2 DNA was used as a template for *in vitro* transcription with a MEGAscript T7 transcription kit (Ambion). The *in vitro*-transcribed RNA was treated with DNase I (Ambion) and then purified by the use of phenol-chloroform-isoamyl acid (Thermo Fisher) and subsequent isopropanol precipitation. Purified RNA was treated with vaccinia capping enzyme (NEB) to generate Cap 0 and, in some cases, also with vaccinia 2′-*O*-methyltransferase (NEB) to generate Cap 1. The quality of the RNA was verified by denaturing agarose gel electrophoresis.

### Transfection.

DNA transfections were performed using PEI MAX (Polysciences, Inc.). *In vitro*-transcribed RNAs were transfected using a TransIT-mRNA transfection kit (Mirus). siRNAs directed against CMTR1 (Dharmacon, Horizon Discovery; catalog no. M-014142-00-0005) or nontargeting siRNA pool 1 (Dharmacon, Horizon Discovery; catalog no. D-001206-13-05) were transfected using Lipofectamine RNAiMax (Invitrogen), and experiments were performed at 48 h posttransfection.

### IFN-β treatment.

All IFN-β (PBL Assay Science) treatments were performed at a concentration of 50 U/ml in cDMEM, unless otherwise noted, for the durations indicated in the figure legends.

### Luciferase assay.

*Gaussia* luciferase activity was measured in supernatants harvested from Huh7-shNT or Huh7-shIFIT1 cells that had been transfected with *in vitro*-transcribed RNA (6 h) after IFN-β treatment (6 h) by the use of a Gaussia Luciferase Flash assay kit (Pierce) according to the instructions of the manufacturer. Samples were read on a BioTek Synergy 2 multimode microplate reader. Data represent averages of results from two technical replicates performed for each biological replicate and were normalized to the luciferase activity of the corresponding Cap 0 RNAs transfected into the shNT cells, which were set to set to a value of 1.

### Focus-forming assay.

Serial dilutions of supernatants were used to infect naive Vero cells in triplicate wells of a 48-well plate. Infected cells were then overlaid with methylcellulose. At 48 h postinfection, cells were fixed in cold 1:1 methanol/acetone and immunostained with anti-flavivirus 4G2 antibody, which recognizes the E protein that was purified in the lab from a hybridoma (1:2,000). Following binding of horseradish peroxidase-conjugated secondary antibody (Jackson ImmunoResearch) (1:1,000), infected foci were visualized with a VIP peroxidase substrate kit (Vector Laboratories) and counted at ×40 magnification. Titers were calculated using the formula (dilution factor × number of foci × 1,000)/volume of infection (μl), with results expressed in units of focus forming units/ml.

### Quantification of viral infection by fluorescence microscopy.

For DENV and ZIKV infections, Huh7 cells treated with siRNAs were infected with DENV or ZIKV (MOI 0.01). At 48 h postinfection, these cells were fixed in 4% paraformaldehyde–phosphate-buffered saline (PBS), permeabilized with 0.2% Triton X-100–PBS, blocked with 3% bovine serum albumin (BSA)–PBS, and then immunostained with anti-flavivirus 4G2 antibody (1:1,000). Infected cells were visualized by the use of Alexa Fluor 488-conjugated secondary antibody (Thermo Fisher) (1:1,000), and nuclei were stained with DAPI (4′,6-diamidino-2-phenylindole; Thermo Fisher). For VSV infections, Huh7 or STAT1^KO^ cells treated with siRNAs were then pretreated with IFN-β (25 U/ml) for 16 h and then infected with VSV-GFP (MOI 0.2). At 8 h postinfection, cells were fixed in 4% paraformaldehyde–PBS and nuclei stained with DAPI. Cells were imaged with a Cellomics ArrayScan VTI microscope at the Duke Functional Genomics Core Facility. The percentage of infected cells was determined by measuring the levels of cells that stained positive for viral antigen relative to the total number of nuclei (10 fields per well, >5,000 cells per condition).

### RT-qPCR.

Total cellular RNA was extracted as described in the associated section below or by using an RNeasy RNA minikit (Qiagen). Then, the RNA was reverse transcribed using an iScript cDNA synthesis kit (Bio-Rad), per the manufacturer′s instructions. The resulting cDNA was diluted 1:5 in water. RT-qPCR was performed in triplicate using Power SYBR green PCR master mix (Thermo Fisher) and a QuantStudio 6 Flex RT-PCR system. Primer sequences are listed in Table 1.

### Immunoblotting.

Cells were lysed in a modified radioimmunoprecipitation assay (RIPA) buffer (50 mM Tris [pH 7.5], 150 mM NaCl, 5 mM EDTA, 0.1% sodium deoxysulfate, 0.5% sodium deoxycholate, and 1% Triton X-100) supplemented with protease inhibitor cocktail (Sigma) and Halt phosphatase inhibitor (Thermo Fisher), and postnuclear lysates were isolated by centrifugation. Quantified protein volumes (7 to 10 μg) were resolved by SDS/PAGE, transferred to nitrocellulose membranes in buffer (25 mM Tris-HCl, 192 mM glycine, 0.01% SDS), and then blocked in a mixture containing 3% bovine serum albumin (BSA; Sigma) with Tris-buffered saline and 0.01% Tween 20 (TBS-T). After washing was performed with phosphate-buffered saline containing 0.01% Tween 20 (PBS-T) buffer or TBS-T buffer (for p-STAT1), membranes were incubated with species-specific horseradish peroxidase-conjugated antibodies (Jackson ImmunoResearch) (1:5,000) followed by treatment of the membrane with Clarity Western ECL substrate (Bio-Rad) and imaging on a Li-COR Odyssey Fc imaging system. The following antibodies were used for immunoblot analysis: rabbit anti-CMTR1 (Atlas Antibodies; catalog no. HPA029980) (1:1,000); mouse anti-ISG15 (Santa Cruz Biotechnology; catalog no. sc-166755) (1:1,000); mouse anti-IFITM1 (Proteintech; catalog no. 60074-Ig) (1:1,000); mouse antitubulin (Sigma; catalog no. T5168) (1:5,000); rabbit anti-MX1 (Abcam; catalog no. ab207414) (1:1,000); mouse anti-IFIT3 (Abcam; catalog no. ab76818) (1:1,000); rabbit anti-IFIT1 (gift of Ganes Sen [17]); mouse anti-pSTAT1 (pY701) (BD; catalog no. 612132) (1:1,000); rabbit anti-GAPDH (anti-glyceraldehyde-3-phosphate dehydrogenase) (GeneTex; catalog no. GT239) (1:1,000); mouse anti-STAT1 (BD; catalog no. 610115) (1:1,000); and mouse anti-FLAG (Sigma; catalog no. F3165) (1:5,000).

### Quantification of immunoblots.

Following imaging using the Li-COR Odyssey Fc imaging system, immunoblots were quantified using ImageStudio Lite software, and raw values were normalized to total protein (Revert 700 total protein stain; Li-COR) for each condition.

### Polysome profiling.

Cells were harvested from 10-cm diameter plates by trypsinization at the indicated time points (0.2 mM; Sigma) and were lysed in lysis buffer (200 mM KCl, 25 mM HEPES [pH 7.0], 10 mM MgCl_2_, 2% n-dodecyl β-d-maltoside [Chem-Impex], 1 mM dithiothreitol [DTT], 40 U RNasin) for 5 min on ice. Postnuclear lysates were isolated and then centrifuged on 15 to 50% sucrose gradients prepared in polysome gradient buffer (200 mM KCl, 25 mM HEPES [pH 7.0], 15 mM MgCl_2_, 1 mM DTT) at 35,000 × *g* for 3.5 h at 4^0^C. Following centrifugation, 12 fractions were collected from each sample using a Bio Comp piston gradient fractionator fitted with a Triax flow cell to measure absorbance. RNA was extracted from each fraction using TRIzol LS (Thermo Fisher), and RNA quality was assessed on a 1% agarose gel. Equal volumes of RNA from each fraction were subjected to RT-qPCR.

### Immunoprecipitation of IFIT1-bound RNA.

Cell extracts were harvested in lysis buffer (100 mM KCl, 5 mM MgCl_2_, 10 mM HEPES [pH 8.0], 0.5% NP-40 supplemented with protease inhibitor cocktail [Sigma] and RNasin RNase inhibitor [Promega]), and lysates were cleared by centrifugation. RNP complexes were immunoprecipitated with protein G Dynabeads (Invitrogen) previously bound with anti-FLAG antibody (Sigma) overnight at 4°C with head-over-tail rotation and were then washed five times in ice-cold NT2 buffer (50 mM Tris-HCl [pH 7.4], 150 mM NaCl, 1 mM MgCl_2_, 0.05% NP-40). Protein for immunoblotting was eluted from 25% of the beads by heating at 95°C for 5 min in 2× Laemmli sample buffer (Bio-Rad). RNA was extracted from the remaining 75% of the beads using TRIzol (Thermo Fisher). Equal volumes of eluted RNA were used for cDNA synthesis, quantified by RT-qPCR, and normalized to RNA levels of input samples.

### Nuclear-cytoplasmic fractionation.

Following siRNA treatment (48 h) and IFN-β treatment (6 h), cells were harvested by trypsinization and lysed in 200 μl lysis buffer (10 mM Tris-HCl [pH 7.4], 140 mM NaCl, 1.5 mM MgCl_2_, 10 mM EDTA, 0.5% NP-40) on ice for 5 min. Following centrifugation at 12,000 × *g* at 4°C for 5 min, the supernatant (cytoplasmic fraction) was collected, and the nuclear pellet was rinsed twice with lysis buffer. RNA was extracted from the cytoplasmic and nuclear fractions using TRIzol and analyzed by RT-qPCR.

### Measurement of mRNA stability.

Following siRNA treatment (48 h) and IFN-β treatment (6 h), cells were treated with 10 μg/ml actinomycin D (Sigma). Lysates were harvested at the indicated time points posttreatment, and RNA was extracted using TRIzol and analyzed by RT-qPCR. Data were normalized as the percentage of RNA remaining at each time point after treatment, relative to that at the time of treatment.

### Statistical analysis.

Statistical tests were performed using GraphPad Prism Version 8.3.0.
